# Contrast-Enhanced Ultrasound: An Effective Method for Noninvasive Diagnosis of Mummified Thyroid Nodules

**DOI:** 10.1155/2022/4289708

**Published:** 2022-04-27

**Authors:** Hong Zhang, Xiaoqu Tan, Linxue Qian

**Affiliations:** Department of Ultrasound, Beijing Friendship Hospital, Capital Medical University, Beijing, China

## Abstract

Mummified thyroid nodules are a special type of thyroid nodule, which is benign, but is often diagnosed as malignant by ultrasound. This study investigated the usefulness of contrast-enhanced ultrasound (CEUS) in the diagnosis of mummified nodules. 66 patients with mummified nodules were divided into two groups: a no-enhancement group and a low-enhancement group. 32 patients with papillary thyroid carcinoma (PTC) were recruited in control group. In the no-enhancement group, CEUS showed that there was no contrast agent entering the nodules, with or without a little dot enhancement or regular ring enhancement around the nodules. The low-enhancement group showed low enhancement inside nodules, which was similar to that in the PTC group. In semiquantitative time-intensity curve analyses, intensity maximum of the central area of nodules in the low-enhancement group was lower than that in the PTC group (*P* < 0.05) and time to peak of the central area of nodules in the low-enhancement group was lower than that in the PTC group (*P* < 0.05). The results demonstrate that CEUS could be used to effectively diagnose mummified nodules, obviating the need for patients to undergo invasive examination such as biopsy or even surgery.

## 1. Introduction

A series of changes may occur in benign cystic thyroid nodules and mixed cystic and solid thyroid nodules, such as absorption of cystic fluid, partial necrosis of solid components, collapse of cystic walls, as well as others. After a period of time, the nodules may become dry, shrink, and solid. Lacout et al. [1] named them mummified nodules to describe this process, which can be spontaneous or artificial, such as after a nodule biopsy or ablation treatment [2, 3]. Mummified nodules often show malignant signs such as being very hypoechoic, being calcified, having irregular margins, and having taller-than-wide shape [4]. The ultrasound parameters are included in the Thyroid Imaging Reporting and Data System (TI-RADS) score, which shows, compared to the single ultrasound features [5]. According to the American College of Radiology Thyroid Imaging Reporting and Data System (ACR TI-RADS), these nodules are often classified as TI-RADS 4 or 5 [6]. If the medical history is not clear, a fine-needle aspiration biopsy (FNAB) or even surgical resection is recommended for such nodules. Obviously, the diagnostic efficiency of conventional ultrasound (US) for mummified thyroid nodules is limited. There have been many studies focused on the application of CEUS for examining thyroid, and experience has been gained in differentiating benign from malignant thyroid nodules. If mummified nodules can be identified using CEUS, patients may be exempted from invasive examinations such as a thyroid biopsy. By reviewing the imaging and pathological results of thyroid nodules in our hospital in recent years, we have summarized and compared the imaging features of mummified nodules and thyroid cancer and explored the significance of CEUS in differentiating mummified nodules. There are many histological classifications of thyroid carcinoma, the most common type of which is papillary thyroid carcinoma (PTC) [7]. Because the use of CEUS has been established for PTC but not for other types of thyroid carcinomas, PTC was chosen to be the control group.

## 2. Materials and Methods

### 2.1. Study Population

This study was approved by the Ethics Committee of Beijing Friendship Hospital, Capital Medical University. Informed consent was not required for this study.

In this study, we found that there were two manifestations of mummified thyroid nodules using CEUS: one was no enhancement inside the nodules and the other was low enhancement inside the nodules. Therefore, in this study, mummified nodules were divided into two groups: no-enhancement group and low-enhancement group, and the control group was the PTC group.

At least one of the following diagnostic criteria for mummified nodules had to be met: (1) nodules had a history of gradually shrinking from typical benign cystic nodules or mixed cystic and solid nodules or (2) black or brown sticky necrotic matter was extracted using an US-guided FNAB, which was diagnosed as benign with cytopathology. The inclusion criteria for the PTC group were having had FNA cytology and a positive BRAF V600E mutation or postoperative histopathology verified. Diagnosis of PTC depends on postoperative pathology.

All patients included in this study were enrolled from January 2016 to December 2020. There were 36 patients with 36 nodules in the no-enhancement group, 30 patients with 30 nodules in the low-enhancement group, and 32 patients with 33 nodules in the PTC group.

### 2.2. US Examinations, CEUS Imaging, and US-Guided FNAB

US examination, CEUS, and US-guided FNAB were all performed by US doctors who have had more than 8 years of working experience. US and CEUS instruments used were Philips EPIQ 5 (Philips, The Netherlands) with L12-5 and eL18-4 linear array probes; Philips IU Elite (Philips, The Netherlands) with L12-5 and L9-3 linear array probes; and Philips IU 22 (Philips, The Netherlands) with L12-5 and L9-3 linear array probes.

Ultrasonography described the target nodules according to ACR TI-RADS, including size, composition, echogenicity, shape, margin, and calcification. Nodule size was defined by the maximum diameter. The composition of nodules was described as solid, cystic, or mixed cystic and solid. The echogenicity of normal thyroid parenchyma was defined as isoechoic, lower than this was hypoechoic, and very hypoechoic was more hypoechoic than strap muscles. A taller-than-wide shape was assessed on a transverse image with measurements parallel to a sound beam for height and perpendicular to it for width. Nodule margin was described as smooth, ill-defined, lobulated or irregular, or extrathyroidal extension. The strong echo in nodules was calcification; calcification <1 mm was described as microcalcification, and >1 mm was described as coarse calcification.

**Figure 1:**
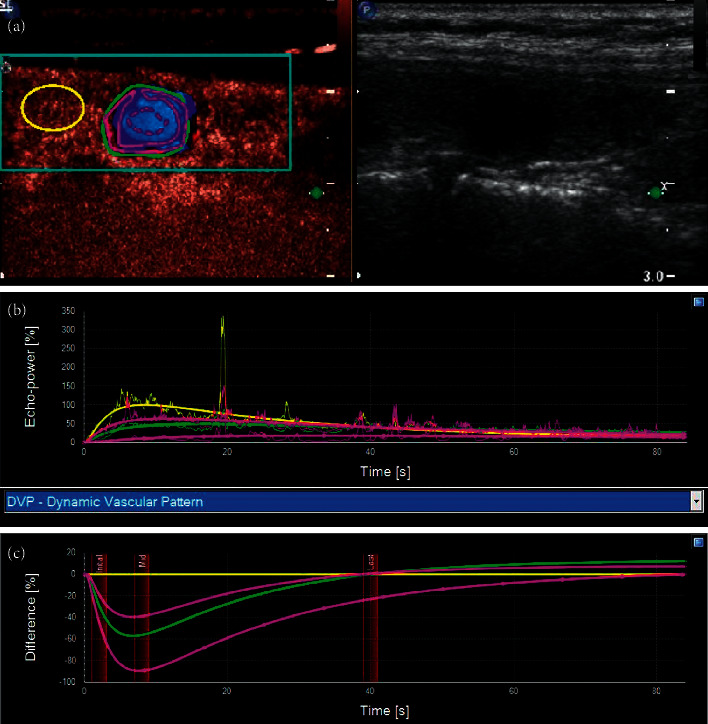
(a) SonoLiver software was used to analyze the dynamic contrast video. A series of regions of interest were demarcated. (b) Time-intensity curve. (c) Time-difference curve.

The standard plane used in the CEUS was the section that showed both a whole nodule and as much of its surrounding thyroid gland as possible. Ultrasond contrast agent (SonoVue®, Bracco International, Milan, Italy) was injected into an antecubital vein with a bolus of 1.6 ml, followed by a 5-ml saline flush. Two-min real-time dynamic video was stored in DICOM format. The enhancement mode, intensity, and margin of nodules after contrast enhancement were observed. SonoLiver (TomTec Imaging Systems GmbH, Munich, Germany) software was used to analyze the dynamic contrast video. First, the target area was demarcated, shown as blue-lined box, which included nodule and periphery thyroid parenchyma. Then, the whole nodule was outlined along the edge of the nodule using green line denoted as Analysis, and the thyroid parenchyma in the same depth was denoted as Reference shown as yellow circle. Attention was paid to avoid blood vessels and calcification. Additionally, two other regions were delineated in the nodule area; one was the annular edge of the nodule, denoted as 1. Parametric, marked as red solid line loop, and the other was the central area of the nodule, denoted as 2. Parametric, marked as red dotted line loop (Figure 1(a)). Time-intensity curve was got after that and also a time-difference curve. Time was shown on the *X*-axis, and the intensity difference between the region of interest and the control area was shown on the *Y*-axis. The difference was positive when the intensity in the nodule was higher than that in the control area and negative when the intensity was lower than that in the control area (Figures 1(b) and 1(c)). Then, several parameters were got from these curves: IMAX (intensity maximum), RT (rise time), TTP (time to peak), MTT (mean transit time), and QOF (quality of fit). QOF was the quality control index that indicated the fitting degree between the delineated region of interest and the nodules. The high fitting degree indicated that the region of interest tracked the nodule well. The reliability of the analysis results was high. It was generally required to be greater than 70%. The contrast IMAX of the periphery thyroid parenchyma was defined as 100%, and the contrast intensity of other areas of interest was compared with it and was also expressed as a percentage.

HI VISION Ascendus (Hitachi, Japan) with L75 linear array probe (8–15 MHz) was used for US-guided FNAB. A 25-G puncture needle was used for FNAB, without any negative pressure in the whole puncture process, and repeated punctures covered as much of the whole nodule as possible. The aspiration was sprayed onto a slide and then was fixed in 95% ethanol. Hematoxylin & eosin staining used in the cytological diagnosis was performed in the pathology department.

### 2.3. Data and Statistical Analysis

Statistical analysis was performed using SPSS 16.0 software (IBM Corp., Armonk, NY, USA). Continuous variables were tested for normal distribution. The data conforming to normal distribution were expressed as the mean ± standard deviation, and an independent samples *T* test was used for comparison between the two groups. Data that did not conform to normal distribution were expressed as the median (interquartile range). An independent sample nonparametric test was used in the non-normal distribution comparison between the two groups. The chi-square test was used to compare the data of the classification variables. *P* < 0.05 was considered statistically significant.

## 3. Results

The basic information for the no-enhancement group, the low-enhancement group, and the PTC group are shown in Table 1.

As described above, there were two diagnostic criteria for mummified nodules: those with a history of shrinking from benign nodules and those in which necrotic substances were extracted from nodules using FNAB and pathologically benign. For the two modes of mummified nodules, there were 6 and 30 cases in the no-enhancement group and 5 and 25 cases in the low-enhancement group respectively (Table 2).

**Figure 2:**
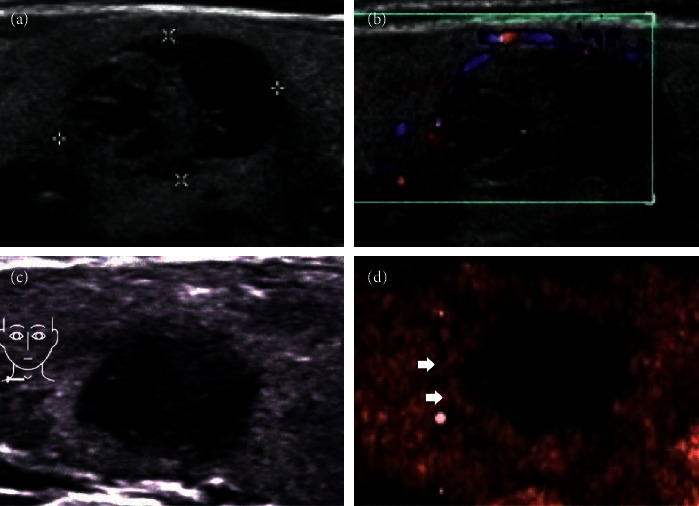
(a, b) A 52-year-old female patient with well-defined cystic and solid nodules in the thyroid gland, and the maximum diameter of the nodules was 17 mm. Color Doppler flow imaging showed blood flow signals in the peripheral and internal solid parts. (c) Three years later, the nodule became smaller, the maximum diameter of the nodule was 9 mm and the margin of the nodule was irregular. (d) CEUS showed no enhancement in the nodules. Short arrows showed annular enhancement at the edges of the nodules. A clear boundary of the nodules could be seen by careful observation.

**Figure 3:**
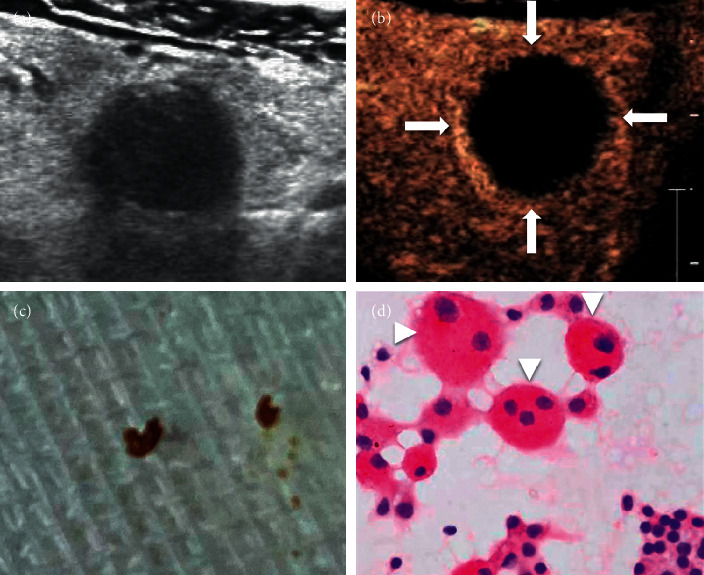
(a) A 63-year-old male, who was found to have a very hypoechoic solid nodule of the thyroid, with ill-defined margin and posterior echo attenuation. It was classified as TI-RADS 4. (b) CEUS showed no enhancement at all in most areas of this nodule, and only the edge of the nodule showed regular annular enhancement with clear margin. (c) The aspirate of the FNAB was a dry brown substance. (d) Multinucleated giant cells and hemosiderin were easily to be seen in cell pathology (x200).

In the no-enhancement group, there was no contrast agent entering the nodules at all, with or without a little dot enhancement or regular ring enhancement around the nodules. Irregular nodule margins in two-dimensional US became clear in CEUS (Figures 2 and 3).

**Figure 4:**
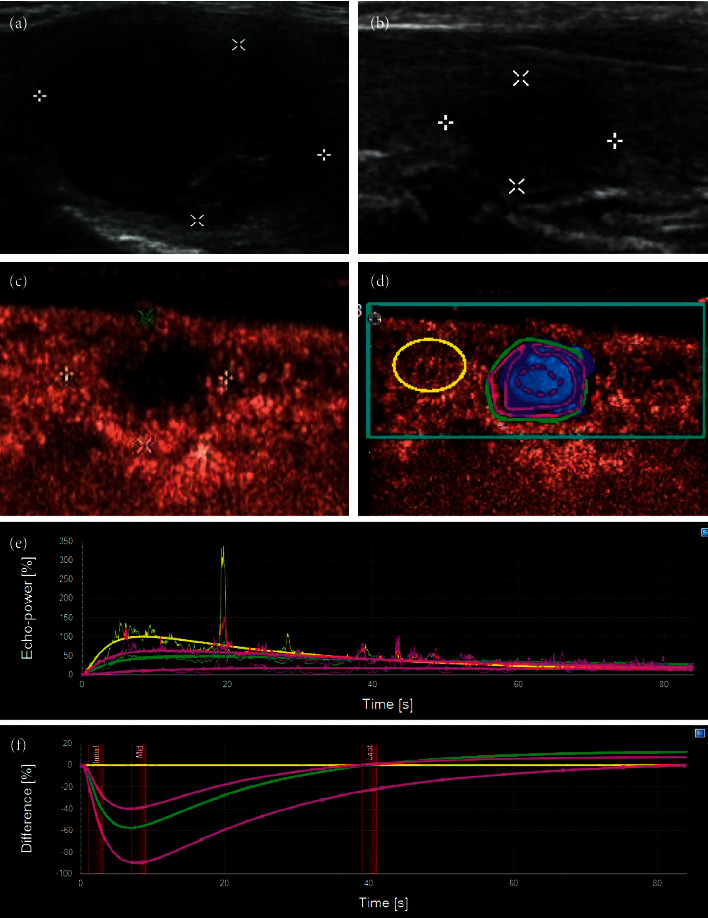
(a) A 53-year-old female had a thyroid nodule with clear margin and the maximum diameter of 21 mm. (b) Two years later, the nodule became smaller and more solid, the margin of the nodule was blurred, and the nodule was classified as TI-RADS 4. (c) CEUS showed that the edge of the nodule was slightly ring-shaped with low enhancement, while the interior of the nodule was uneven with low enhancement. (d) The contrast video was analyzed using SonoLiver software. (e, f) The time intensity curve and time-difference curve were obtained. It showed that the intensity of the whole nodule, its periphery, and its interior was always lower than those in the control area with time, and the intensity in the central area was the lowest, which was close to no enhancement.

**Figure 5:**
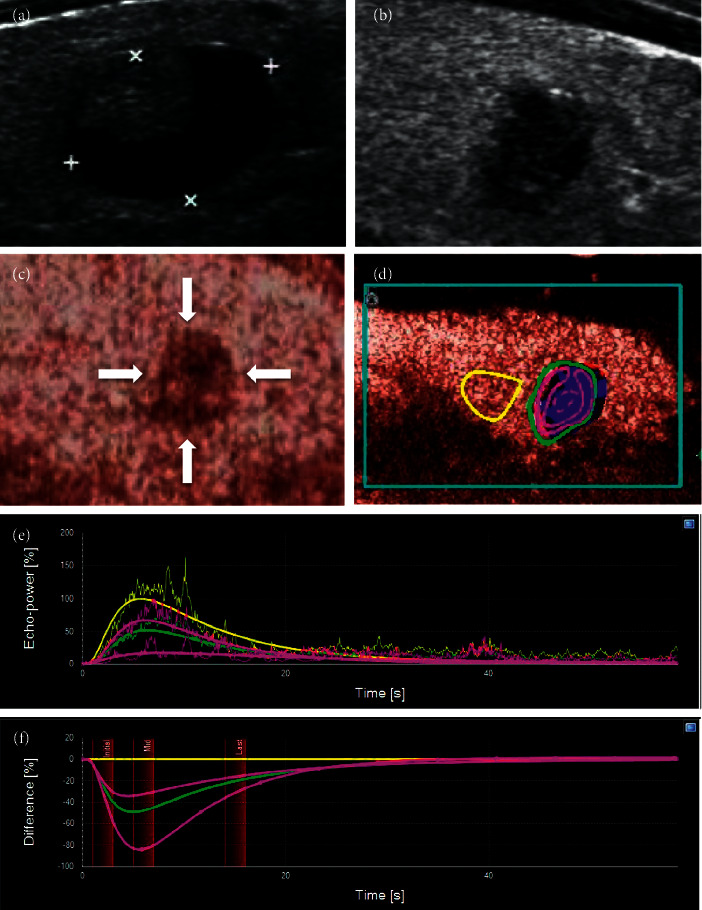
(a) This thyroid nodule had clear margin, and its maximum diameter was 15 mm, which was classified as TI-RADS 3. (b) Five years later, the patient reexamined the thyroid and was found that the nodule had become smaller and more solid, with irregular margin, microcalcifications at the edge, and a taller-than-wide shape, which could be classified as TI-RADS 5. (c) CEUS showed that the contrast mode was enhanced from the periphery to interior. It was uneven with low enhancement, and the margin was not very clear, so it was difficult to distinguish it from a PTC. (d–f) SonoLiver software was used to analyze the contrast video, and the time intensity curve conformed to the performance of mummified nodules.

**Figure 6:**
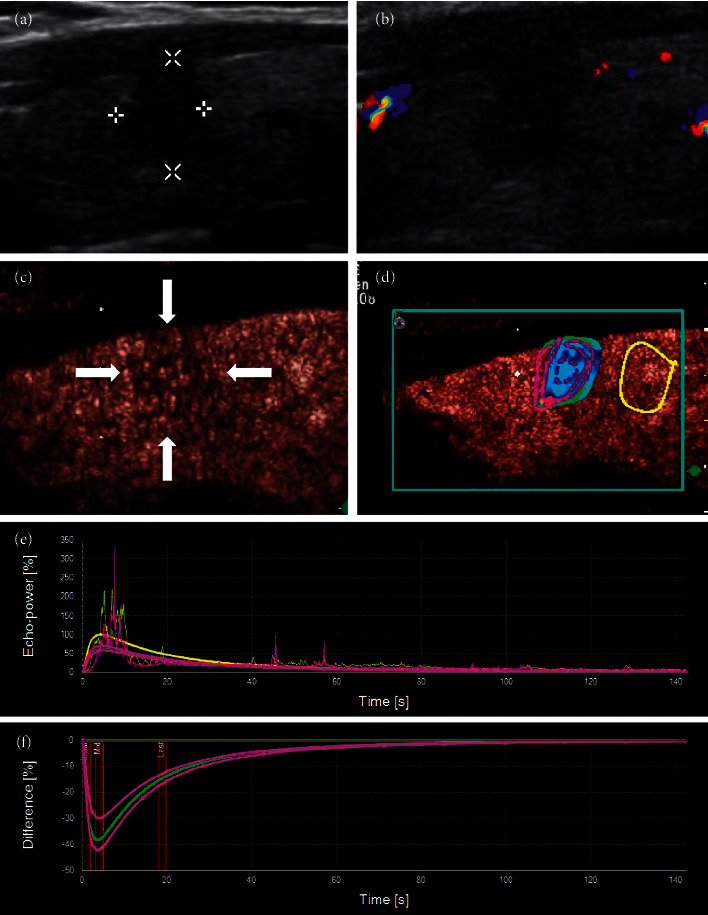
(a, b) A 33-year-old male was found to have a solid nodule with very low echo near to the front capsule of the thyroid. The nodule had irregular margin and a taller-than-wide shape. The discontinuity of anterior capsule indicated extrathyroidal invasion, and no blood flow signal was detected in the nodule. The nodule was classified as TI-RADS 5. (c) CEUS showed that the whole nodule had uneven low enhancement and unclear margin, but had no ring enhancement. (d–f) SonoLiver software was used to analyze the contrast video. According to the analysis results, the nodule was considered to be a PTC. Postoperative pathology confirmed this result.

In the low-enhancement group, a small amount of contrast agent entered inside the nodules, and there was uneven low enhancement compared with the thyroid parenchyma (Figures 4 and 5). This manifestation was similar to the contrast enhancement seen in PTC of the same size (Figure 6). Comparison of contrast analysis parameters between the two groups is shown in Table 3. The IMAX of the whole nodules was lower than that of the thyroid parenchyma in both groups. The IMAX around the nodules was greater than that of the central areas, and there was no significant difference between the two groups. The IMAX of the central area of nodules in the low-enhancement group was significantly lower than those in the PTC group. The TTP of the central area of nodules in the low-enhancement group was longer than those in the PTC group. There was no significant difference in RT or MTT between the two groups.

## 4. Discussion

**Figure 7:**
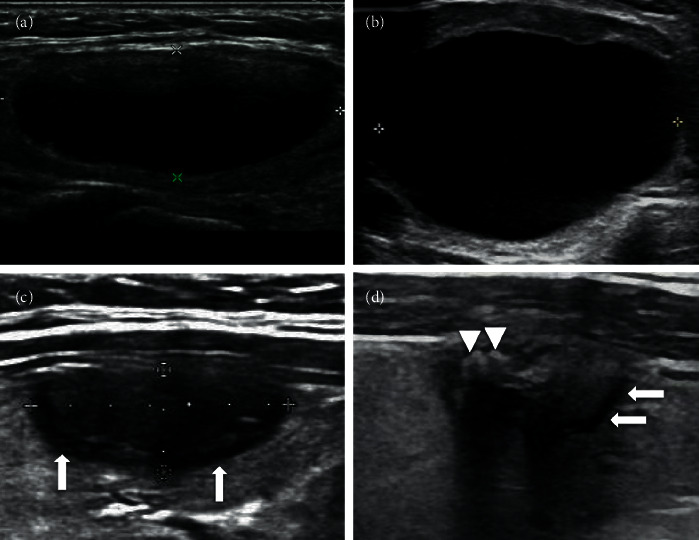
(a) A 44-year-old female was found a thyroid cystic nodule in a health examination, and it was classified as TI-RADS 2. (b) 1.5 years later, the patient felt neck pain, and also a mass with palpation. Ultrasound examination showed that the cyst was enlarged with the maximum diameter 40 mm, and dense punctate echoes could be seen in the capsule cavity, which was considered as hemorrhage in the cyst. (c) 2 years later, ultrasound showed that the nodule shrank and became solid inside, and the maximum diameter of the nodule was 16 mm. Arrows show a hypoechoic halo around nodules, and the margins of nodule were still clear. (d) 3 years later, the nodule was even smaller, and the hypoechoic halo around the nodule was still visible (arrows), but the margin became blurred. The triangle pointed to coarse calcifications, suggesting that necrotic substances exist for a long time. Echo attenuation behind the nodule suggested that fibrosis in the nodule might be serious.

**Figure 8:**
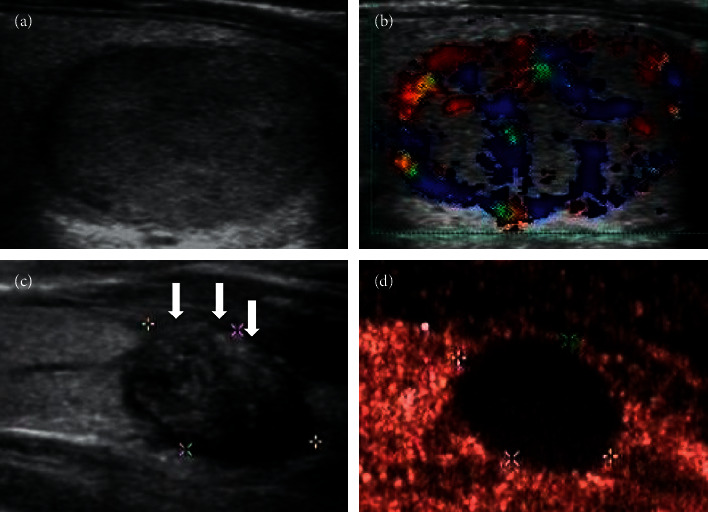
(a, b) A 23-year-old female had an oval solid nodule in the thyroid. The nodule had clear margin and abundant peripheral and internal blood flow signals. The nodule was classified as TI-RADS 3 and considered to be a follicular adenoma. (c) The patient received ultrasound-guided microwave ablation. 1 year after treatment, the nodule became smaller. The anterior capsule of the thyroid was interrupted because of thermal ablation. (d) CEUS showed no enhancement in the nodule suggesting complete necrosis.

Some benign cystic thyroid nodules and mixed cystic and solid thyroid nodules may change in form after a period of time. The causes of the changes are spontaneous absorption of cystic fluid, spontaneous hemorrhage reabsorption in the cystic cavity, and hemorrhage reabsorption after FNAB, as well as others [8]. Nodules have different morphologies associated with the different stages of change. The nodule volume may be enlarged during the stage of intranodular hemorrhage. At the same time, patients can touch obvious thyroid nodules, and sometimes, this is accompanied by pain. Subsequently, the cystic fluid or blood in the nodule is further absorbed and dried, and at the same time, the solid components become necrotic, which leads to the reduction and consolidation of the nodule volume [9], which appears very hypoechoic on US, and the long-term existence of necrotic substances leads to calcifications, which may appear inside or around the nodule [10]. At the same time, the decrease of tension in the nodule causes the capsule wall to collapse, which leads to the irregular margin between the nodule and the surrounding parenchyma. The typical manifestation is the existence of a “double ring sign” [11]. The inner hyperechoic ring may be the collapsed capsule wall, and the outer hypoechoic ring may be fibrous capsule or inflammatory reaction zone [2]. CEUS can show that the outer ring of the nodule is the location with the greatest blood supply. Nodules that are very hypoechoic have calcification, and irregular margins are all signs of thyroid malignant nodules, so these nodules are often classified as TI-RADS 4 or 5 (Figure 7). In addition to the spontaneous changes in nodules, necrosis of benign thyroid nodules happens after ablation treatment. Similar manifestations can be observed after absorption and reduction of nodules (Figure 8).

Some researchers named these types of nodules mummified nodules and summarized the characteristics of these nodules. They found that these benign mummified nodules had malignant shapes after dehydration and drying. If the process of nodule changes can be traced, then diagnosing mummified nodules becomes much easier. If the patient has no definite medical history and lacks the records of previous US examinations, then the manifestation of this kind of nodule will lead to malignant diagnosis. To date, FNAB remains the gold standard technique in the evaluation of both palpable and nonpalpable thyroid nodules [4, 12–14]. The FNAB of the nodule might show some very thick or dry dark substance, which corresponds to the old necrotic substance in the nodule. Pathology will show hemosiderin, fibrosis, and more multinucleated giant cells (Figures 3(c) and 3(d)).

**Figure 9:**
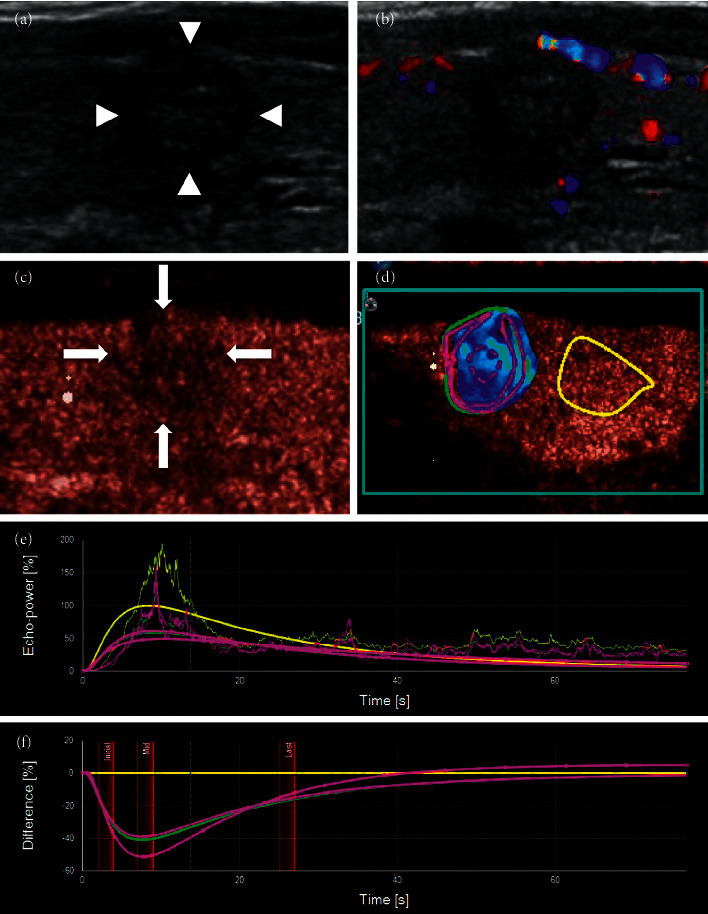
(a, b) A 55-year-old female had a hypoechoic solid nodule in her thyroid, with a maximum diameter of 6 mm and a hypoechoic halo around the nodule, uneven in thickness. The margin of the nodule was not clear, and no blood flow signal was detected in the nodule. The nodule was classified as TI-RADS 4 and was considered to might be a mummified nodule. (c) CEUS showed that the nodule had uneven low enhancement, unclear margin, and no ring enhancement, which was similar to the enhancement mode of a PTC. (d–f) SonoLiver software was used to analyze the contrast video. According to the analysis results, the nodule was considered to be a PTC. Postoperative pathology confirmed this result.

Although pathology is the gold standard for diagnosing benign and malignant nodules, if there is a noninvasive examination that can diagnose mummified nodules, patients can avoid the pain of FNAB or surgery [15]. In recent years, US evaluation of thyroid nodules has been greatly improved with the introduction of new US software, such as CEUS and US-elastography [16]. CEUS displays blood flow by injecting a contrast agent into the peripheral vein. The contrast agent used in this study was SonoVue, which is a pure blood pool contrast agent. The effective components are microbubbles formed by the sulfur hexafluoride inert gas trapped in the lipid shells whose volume is equivalent to that of red blood cells. The microbubbles vibrate under the action of sound pressure to generate harmonic signals that can be captured by probes and form real-time harmonic images. Color Doppler shows the nodules with a lack of a blood supply, which may suggest that the blood vessels inside the nodules are scarce or missing. It is also possible that the blood vessels are small and the blood flow is extremely slow, which leads to the failure of the Doppler display [17]. CEUS can give us more information about microvessels and can be used to evaluate the blood supply of nodules more completely [18]. Moreover, different nodules show different perfusion and regression patterns because of the different density, number, diameter, blood flow rate, and distribution ratio of arteries and veins. Some nodules have similar histological structure, so the CEUS mode will be similar, which imposes rules to follow. In recent years, there have been many studies on PTC using CEUS [18–22]. Small PTCs, especially PTCs less than 1 cm, often show low enhancement on CEUS, suggesting sparse microvessels in the nodules (Figures 6 and 9) [23]. This may be because of the destruction of some blood vessels in the nodules, the complete or incomplete formation of blood vessel beds in early tumors, or the formation of microthrombosis in blood vessels [24, 25].

Through this study, we found that there were two manifestations of CEUS in mummified nodules: one was no enhancement in nodules and the other was low enhancement. No enhancement in nodules indicates that nodules are inactive, and we can be very sure of this diagnosis. The periphery of nodules can show low enhancement, equal enhancement, or slight hyperenhancement. It reflects the state of inflammatory reaction around nodules (Figures 2 and 3). Another manifestation is low enhancement in nodules, which means that the nodules are not completely necrotic (Figures 4 and 5). The typical manifestation of CEUS in small PTCs is uneven low enhancement in nodules, with the low-enhancement area being smaller than the size of two-dimensional US-measured nodules, and the boundary between nodules and thyroid parenchyma is unclear after contrast enhancement. Similarly, the manifestation of low enhancement in nodules makes it difficult to distinguish them from PTCs, especially when the nodules are small, which makes the diagnosis more difficult. Of course, some PTC nodules may also have a hypoechoic halo. So, hypoechoic halo is not unique for mummified nodules (Figure 9).

SonoLiver analysis software can be used to trace the time intensity curves of nodules and surrounding thyroid parenchyma, and the contrast intensity can be semiquantified. From the curve calculation, no significant difference was found in the peak intensity of the whole nodule between the low-enhancement group and the PTC group. However, after analysis, it was found that the peak intensity of the nodule center in low-enhancement group was lower than that in PTCs. This is significant for the identification of the nodules between the two groups. Mummified nodules undergo the process of node shrinkage and necrosis, and most of the nodules are made up of necrotic substances, even if they are enhanced, the intensity is weak. The peak intensity around the nodule is slightly higher in the low-enhancement group than in the PTC group, which may be because of the inflammatory reaction around the mummified nodules. Therefore, the blood supply was slightly richer than that in PTCs, but there was no statistical difference.

Another significant index reflected in the CEUS is TTP. Like most cancers, the more invasive the PTC is, the more nourishing are the arteries to it. The generation of nourishing arteries is reflected in the CEUS, which shows a pattern of enhancement and early rapid enhancement, so the TTP is short. The nature of mummified nodules is benign nodules without large nutrient arteries, which is caused by necrosis and shrinkage of cystic nodules or cystic solid nodules, resulting in a poor blood supply. The contrast enhancement speed is “slow,” which is not obvious to the naked eye. The exact TTP can be obtained by using contrast analysis software. By comparison, it was found that the TTP in in low enhancement group was longer than that in the PTC group. Although the difference was obvious only in the central area of the nodules, this index can still reflect the structural characteristics of nodules, which is helpful for the diagnosis of benign and malignant tumors.

It is worth emphasizing that if CEUS findings are not typical, FNAB is still the gold standard for the diagnosis of mummified nodules. In recent years, molecular detection as a supplement to cytological diagnosis has been applied to clinical operation [5]. Molecular detection is of great significance in the diagnosis, clinical treatment, and prognosis of thyroid nodules. The BRAF^V600E^ mutation is the most prevalent genetic alteration (up to 80%) found in PTC tissues [26]. Mummified nodules are typically benign, and BRAF^V600E^ mutation should be negative. FNAB combined with molecular detection can help reduce the resection rate of mummy nodules.

## 5. Conclusions

In summary, CEUS of mummified nodules has its own characteristics. CEUS can provide more information for diagnosing nodules and differentiating between mummified nodules and PTCs, which can help patients avoid the need for an invasive examination such as biopsy or even surgery.

## Figures and Tables

**Table 1:** Basic information of each group and basic characteristics of nodules.

Group	Tirads		Age (y), median (interquartile range)	Gender, male/female	Nodule size (mm), median (interquartile range)
	4	5			
Nonenhancement group (*n* = 36)	36	0	56 (50,65.75)	15/21	9 (7,10.75)
Low-enhancement group (*n* = 30)	29	2	55 (42.25,59.25)	6/24	8 (6.75,10.25)
PTC group (*n* = 33)	31	2	51 (35.50,59.50)	4/29	9 (7.5,14)
*P* ^*∗*^	0.612		0.337	0.393	0.089

^*∗*^Comparison between the low-enhancement group and the PTC group.

**Table 2:** Diagnostic methods used in the diagnosis of mummified nodules.

Group	Diagnosis of history	FNAB visual judgment and pathological diagnosis
Nonenhancement group (*n* = 36)	6	30
Low-enhancement group (*n* = 30)	5	25

**Table 3:** Comparison of CEUS parameters between the low-enhancement and the PTC groups.

Parameters		Low-enhancement group	PTC group	*P*
IMAX (%), median (interquartile spacing)	Reference	100.00 ± 0.00	100.00 ± 0.00	1.000
	Analysis	46.82 (29.56,64.71)	52.46 (28.16,78.94)	0.731
	1. Parametric	67.88 (48.53,78.65)	52.83 (36.82,94.47)	0.356
	2. Parametric	20.98 (11.23,31.64)	37.90 (21.33,71.76)	0.012

RT (s), median (interquartile spacing)	Reference	5.17 (3.50,7.59)	4.96 (3.50,7.62)	0.611
	Analysis	5.47 (4.09,8.25)	5.65 (3.71,7.68)	0.531
	1. Parametric	5.32 (4.00,7.87)	5.46 (3.69,7.10)	0.601
	2. Parametric	8.07 (4.42,13.08)	6.05 (4.23,8.91)	0.137

TTP (s), median (interquartile spacing)	Reference	6.49 (4.53,8.60)	6.85 (5.43,8.21)	0.620
	Analysis	7.39 (4.46,9.57)	6.52 (5.31,8.33)	0.339
	1. Parametric	7.39 (4.46,8.85)	6.54 (5.18,7.90)	0.335
	2. Parametric	8.62 (5.31,13.93)	6.70 (5.22,9.30)	0.036

mTT (s), median (interquartile spacing)	Reference	20.28 (9.34,51.35)	30.49 (10.06,92.11)	0.205
	Analysis	22.63 (14.65,58,58)	28.61 (11.81,105.99)	0.620
	1. Parametric	20.72 (11.86,43.12)	28.54 (11.97,95.40)	0.441
	2. Parametric	40.00 (22.29,109.72)	44.08 (21.49,101.44)	0.851

## Data Availability

The data used to support the findings of this study are available from the corresponding author upon request.
